# Incidence of varicella in children in Jeju-do, Korea, 2005-2016: age-period-cohort analysis

**DOI:** 10.4178/epih.e2018054

**Published:** 2018-11-08

**Authors:** Jinhee Kim, Ji-Eun Kim, Jong-Myon Bae

**Affiliations:** 1 Jeju Center for Infection Control, Jeju, Korea; 2 Department of Preventive Medicine, Jeju National University School of Medicine, Jeju, Korea

**Keywords:** Chickenpox, Chickenpox vaccine, Cohort effect, Immunization programs, Immunization schedule, Measlesmumps-rubella vaccine

## Abstract

**OBJECTIVES:**

Although the nationwide inoculation rate of varicella vaccine was approximately 95% in Korean children recently, the number of notified varicella cases is unexpectedly continuously increasing till now. To suggest some hypotheses regarding this discrepancy, an age-period-cohort (APC) analysis as a descriptive epidemiology study was conducted for children residing in Jeju-do, Korea.

**METHODS:**

The raw data were obtained from the nationwide database for insurance claim of healthcare fee provided by the National Health Insurance Service, Korea. The selection criteria were children aged 2-13 years who visited any healthcare center due to varicella from 2005 to 2016 while residing in Jeju-do. After calculating the birth cohort-specific crude incidence rates by age and year, the intrinsic estimator method was used to perform the APC analysis.

**RESULTS:**

As the annual crude incidence rates decreased with increasing age between 2005 and 2016, the age and period effects also decreased. The intrinsic estimator coefficients suggesting the cohort effect shifted from positive to negative in 2011, the starting year of free varicella vaccine program in Jeju-do.

**CONCLUSIONS:**

The results suggested that inoculated varicella vaccines have preventive effects. However, further studies to evaluate waning immunity would be needed.

## INTRODUCTION

Since the World Health Organization (WHO)’s recommendation of varicella vaccinations in 1998 [1], the Republic of Korea (hereafter Korea) included varicella vaccination in the list of national essential vaccine requirements in January 2005 and has administered one round of varicella vaccine to infants and toddlers aged 12-15 months [2]. According to the Korean Statistical Information Service (http://kosis.kr), varicella vaccination rate in Korea in 2016 reached 97.5% [3]. A pre-market clinical trial found that one round of varicella vaccination produces antibodies that protect against 95% of varicella [4], and post-market vaccine effectiveness (VE) was reported to range from 80 to 85% [5,6]. Furthermore, a 10-year follow-up after vaccination revealed that VE is maintained in 94.4% of children after one round of vaccination [7].

Despite the expectations of markedly lower varicella infections in Korea with such high VE and vaccine rate, the number of reported varicella cases in the Web-based Infectious Diseases Statistics System (http://is.cdc.go.kr) maintained by the Korea Centers for Disease Prevention and Control (KCDC) increased by 2.7 times from 20,284 cases in 2007 to 54,060 cases in 2016 [2], and breakthrough varicella infection has been reported to occur among vaccinated children [8-10]. Although the varicella vaccination rate in Jeju-do, which has been providing free varicella vaccinations to children aged 12-15 months since 2011, reached 97.0% in 2015-2016 [3], the number of varicella infections increased by 125% in 2018 compared to that in the same period in 2017 [11], and an epidemiologic investigation of varicella found that all of the infected patients had been vaccinated [12].

Descriptive epidemiological studies are needed in order to develop a valid explanation for the increasing incidence of varicella infection despite implementation of varicella vaccination projects. Therefore, this study aimed to develop a hypothesis via an age-period-cohort (APC) analysis of varicella infections among children in Jeju-do.

## MATERIALS AND METHODS

Raw data were obtained from the customized database (DB) in the health insurance big data provided by the National Health Insurance Service (NHIS; http://www.nhis.or.kr). This is a nationwide DB created based on the claim data submitted to the NHIS by health facilities nationwide.

The inclusion criteria were (1) assigned Korean Standard Classification of Diseases codes B01-B01.9 as the main and nine of the additional disease codes between 2005 and 2016; (2) residents of the Jeju-do; and (3) children aged 2-13 full years at the time of claim. The first year of observation was set to 2005 in consideration of the fact that varicella vaccine was designated as a required vaccine in that year. The minimum age was set to 2 because Korea’s varicella vaccine guideline indicates that the vaccine should be administered within 12-15 months after birth, and maximum age was set to 13 because varicella infection occurs in elementary school or younger patients. Furthermore, when the varicella code was claimed more than once in a year for the same patient, the remaining codes were considered continuous treatment for a past varicella infection and were excluded.

Crude incidence rate (CIR; per 1,000 persons) by year and age was defined as the number of claims obtained from the DB divided by the mid-year population for the same age and year, respectively, obtained from the Statistics Korea (http://kosis.kr).

The intrinsic estimator (IE) method was used for APC analysis [13], as this method was developed to address the linear dependency among APC because the cohort is determined according to age and period [14]. The *<apc_ie>* package provided by the Stata SE version 14 (StataCorp., College Station, TX, USA) was used for IE application. This study was exempted from institutional review board, as secondary data excluding personally identifiable information are used.

## RESULTS

**Figure 1.**
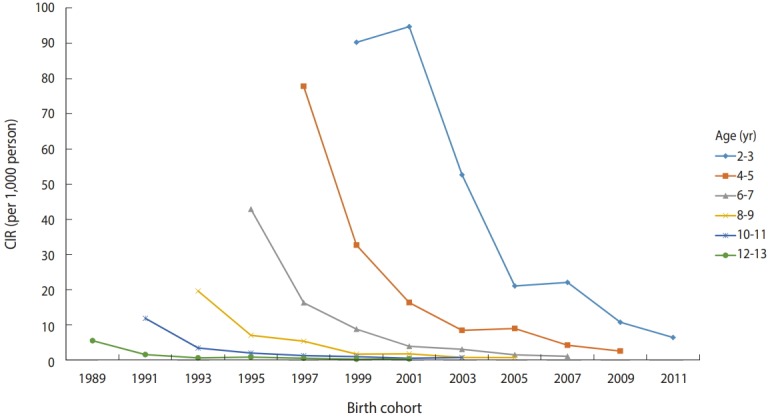
The birth cohort-specific crude incidence rates (CIR, per 1000 person) of varicella according to age group in children in Jeju-do, Korea.

Table 1 shows the CIRs of varicella among children aged 2-13 years in 2005-2016. CIR decreased with increasing age in all years. In the period 2005-2010, prior to the introduction of free primary vaccination, CIR tended to decrease as the recent years increased in all ages. After 2011, the decreasing trend was still evident among children aged 2-6 years, while showing no decreasing or actually increasing trend among children aged ≥7 years. Figure 1 shows the CIR graph of birth cohorts by age group. CIR tended to decrease with more recent birth cohorts in the same age group.

**Figure 2.**
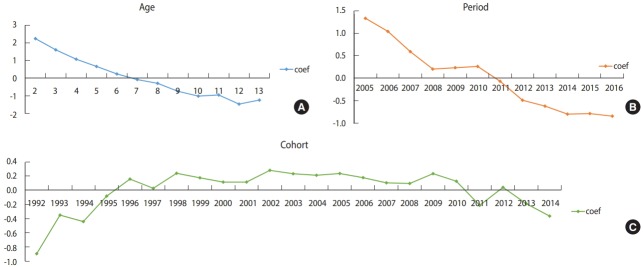
Intrinsic estimator (coef) of (A) age, (B) period, (C) cohort effect from annual age-specific crude incidence rates (1,000 person) of varicella by age (2-13 years old) and calendar year (2005-2016) in children in Jeju-do, Korea.

Figure 2 shows the results of age, period, and cohort effects on CIR of varicella among children aged 2-13 years in 2005-2016. In support of previous interpretations, varicella incidence decreased with increasing age after adjusting the period and cohort effects, and also decreased with more recent years after adjusting the age and cohort effects. Furthermore, regarding cohort effects, the risk for varicella tended to decrease among children born on 2012 or later after adjusting the age and period effects.

**Figure 3.**
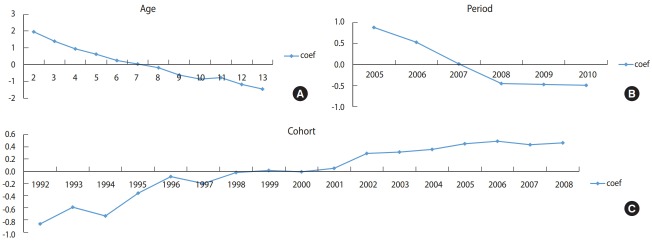
Intrinsic estimator (coef) of (A) age, (B) period, (C) cohort effect from annual age-specific crude incidence rates (1,000 person) of varicella by age (2-13 years old) and calendar year (2005-2010) in children in Jeju-do, Korea.

Figure 3 shows the results of APC analysis for CIR of varicella in 2005-2010, which is before introduction of free varicella vaccination in Jeju-do. Varicella incidence tended to decrease with increasing age and decreased from 2005 to 2008, after reaching a plateau. On the contrary, varicella incidence consistently increased from the 1992 to 2008 cohort.

**Figure 4.**
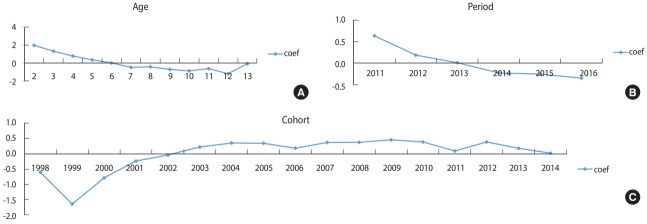
Intrinsic estimator (coef) of (A) age, (B) period, (C) cohort effect from annual age-specific crude incidence rates (1,000 person) of varicella by age (2-13 years old) and calendar year (2011-2016) in children in Jeju-do, Korea.

Figure 4 shows the results of APC analysis for CIR of varicella in 2011-2016, the period after introducing the free varicella vaccination in Jeju-do. Varicella incidence tended to decrease with increasing age, and also from 2011 to 2016. It also tended to decrease in cohorts after the 2012 cohort.

## DISCUSSION

In summary, the risk for varicella decreased with increasing age, and in more recent years, the risk for varicella decreased among cohorts born after 2011, the year of launching free varicella vaccination. In other words, based on the NHIS data, varicella incidence decreased from 2005 to 2016 among children in Jeju-do, supporting that the varicella vaccine is indeed effective.

Based on the results confirming the effectiveness of varicella vaccines, we can infer a few things. First, if we continue the vaccination project per the vaccination guidelines and achieve high vaccination rates, we can predict that varicella incidence will decrease by the year 2025, the year in which children born on 2011 reach the age of 14. This is under the premise that the risk for varicella decreases with increasing age. Second, amid current concerns about vaccination failure and ineffective vaccines due to the rising varicella incidence even after vaccination, these results serve as evidence supporting that varicella vaccines administered to children in Jeju-do are indeed effective in preventing varicella infection. This is based on the results of APC analysis for 5 years of data since 2011, the first year of free vaccination.

Regarding the continuous increase of reported varicella infection cases in Jeju-do despite the evidence supporting the effectiveness of varicella vaccines, we can infer the following. First, this phenomenon may be attributable to the gap between the number of claims made to the NHIS, the source of our data, and the number of cases reported in the annual report of varicella monitoring by the KCDC. The gap between these two sources of data tends to increase as we move further in the past and decrease in more recent years [2]. Thus, the KCDC report of varicella cases suggests that it has been rapidly rising, but the NHIS claim data suggest that varicella infections have been decreasing. Second, the increasing number of varicella infections among children in Jeju-do as of today in 2018 may be due to the relatively higher incidence among children born before 2011, the first year of free varicella vaccination. This is based on the fact that since 2011, Jeju-do has been giving mandatory varicella vaccination to children aged 12-15 months of birth per the national vaccination guideline, and the vaccination rate is >97%.

Furthermore, we need to develop valid hypotheses for the breakthrough of varicella infections affecting vaccinated children despite the evidence supporting the effectiveness of varicella vaccines [8-12]. Breakthrough of varicella occurrence can be interpreted as a result of secondary vaccine failure, in which an immunity acquired via varicella vaccine administered within 12-15 months of birth is not maintained over time, as opposed to the primary vaccine failure in which no immunity was acquired at all in the first place. Evidences supporting this hypothesis are as follows [1]. First, a cross-sectional, seroepidemiological study found that the seroprevalence of varicella virus was 75% among 1-2-year-olds but decreased to 57.5% among 3-5-year-olds in serum samples collected in 2009-2010 in Korea [15]. Second, case-control studies reported that the VE of 5-year-old children born in Korea who were administered varicella vaccination before 15 months old were 54% [8] and 13.0% (95% confidence interval [CI], -17.3 to 35.6) [16]. Third, a cohort follow-up of American children found that the incidence of breakthrough varicella increases over time after vaccination [17]. Based on this report, the US has inoculated secondary vaccination in children aged 4-6 since 2006 [18,19].

One study has shed light on the possible cause of the need to administer secondary vaccination due to secondary vaccine failure. In a retrospective cohort study published in 2003 [20], the risk for breakthrough varicella increased by 3.1 times (95% CI, 1.5 to 6.4) when varicella vaccine was administered within 28 days after administering the attenuated measles-mumps-rubella (MMR) vaccine. This serves as grounds emphasizing compliance to the principles of vaccination, in which attenuated vaccines must be administered with 4-week intervals in order to lower the interference of immunogenicity between two different live vaccines [2]. Although the said study did not provide additional data on concurrent vaccination, based on the description regarding the computation of vaccination intervals indicated in the Materials and Methods section, it might be likely that they included those who received concurrent vaccination, that is, those who had a 0-day interval between the two vaccines, into the breakthrough varicella risk group.

The measles-mumps-rubella-varicella (MMRV) vaccine was commercially approved in 2009 based on the evidence of its noninferiority in terms of immunogenicity and efficacy to concurrent vaccination of two vaccines (MMR+MMRV) [21], which is currently administered to children in the US, Germany, Australia, Italy, and India [20-24]. The Korean vaccination guideline also recommends concurrent vaccination of the primary MMR and varicella vaccine based on its benefit of increasing the vaccination rate and timely vaccination [2]. In summary, vaccination projects recommend concurrent vaccination without results from comparative effectiveness research that compares the VE of three head-tohead vaccination methods: concurrent MMR+MMRV, MMR vaccination after 4 weeks of MMRV vaccination (MMRV>MMR), and MMRV vaccination after 4 weeks of MMR vaccination (MMR>MMRV).

Nonetheless, measles virus infection is known to inhibit the cellular immunity. *In vitro* studies have found weakening of the cellular immune system, such as lower concentrations of interleukin and interferon, which are produced by mononuclear cells, and absence of delayed hypersensitivity over several weeks [25-28], and studies have also reported that such abnormal cellular immune responses temporarily occur after measles vaccination [28-30]. Then, the long-term VE of the current method of concurrent MMR+MMRV vaccination and sequential MMRV>MMR vaccination should be investigated. To obtain such evidence, a sequential vaccination schedule, in which varicella vaccine is first administered at 12-13 months after birth and the first MMR vaccine is administered 4 weeks later, at 14-15 months after birth, which still complies with the national vaccination guideline, could be implemented. Then, a prospective cohort study should be conducted on children who were administered these vaccines concurrently and those who were administered these vaccines sequentially to identify the risk of breakthrough varicella among them. If results indicate that the MMRV>MMR vaccination method leads to better immunogenicity of varicella vaccine, no secondary vaccination would be required. Therefore, epidemiological studies should be conducted from multiple angles to investigate the long-term preventive effects of varicella vaccines according to various vaccination schedules in children in Korea.

This study has few limitations. First, we used the claim data for medical treatment, as opposed to definitive diagnosis based on serological testing, to define varicella; therefore, patients with suspected varicella could also have been included. However, considering that varicella is diagnosed based on the clinical examination, as opposed to laboratory tests, using the claim data seems valid. Second, we could not distinguish breakthrough varicella because the data only specified the number of varicella cases. However, considering the high vaccination rate among children born after 2011, the first year of free varicella vaccination, most varicella claims could be considered as cases of breakthrough varicella. Interpretation of results for children born on and before 2010 should be made in consideration of this fact. Third, children who have minimal clinical symptoms from breakthrough varicella may have not visited a healthcare facility. If so, the CIR shown in our data may be underestimated. However, our interpretation of APC results would be still valid if this notion would be similar across ages and years. Last, we included main disease code and nine additional disease codes in the claim data when defining cases of varicella; however, CIR may differ according to the inclusion criteria. However, this study aimed to analyze the APC effects, as opposed to computing the absolute CIR; hence, maintaining a consistent criterion would be more important. In the future, studies should analyze sensitivity to establish a valid standard in defining varicella from NHIS data.

In conclusion, we found the effects of period, in which varicella incidence decreased in more recent years since 2005, as the year in which varicella vaccination was designated a legally required vaccine and was recommended), and a cohort effect, in which risk for varicella decreased among children born after 2011, the first year of free varicella vaccine. Therefore, it is safe to claim that the current varicella vaccine is effective in preventing varicella infection. However, further study is needed to compare the effects according to vaccination schedule in order to develop grounds for the long-term effects of the vaccine and to address the relevant issue, we suggest a sequential vaccination schedule in which MMR vaccine is administered 4 weeks after administration of varicella vaccine to infants aged 12 months.

## Figures and Tables

**Table 1.** Annual age-specific crude incidence rates (CIR)^1^ and numbers of varicella cases by age (2-13 years) and calendar year (2005-2016) in children in Jeju-do, Korea

Age (yr)	Calendar year											
	2005	2006	2007	2008	2009	2010	2011	2012	2013	2014	2015	2016
2	95.55 (664)	69.69 (453)	45.31 (294)	26.60 (167)	25.37 (166)	27.36 (177)	24.61 (148)	13.85 (82)	9.62 (62)	9.52 (68)	7.17 (48)	5.86 (41)
3	53.23 (400)	36.25 (247)	21.09 (147)	15.69 (104)	16.79 (103)	16.86 (113)	11.10 (70)	7.66 (47)	7.19 (43)	3.27 (23)	4.67 (33)	3.91 (28)
4	25.23 (216)	20.54 (155)	13.44 (97)	9.55 (67)	11.35 (76)	12.46 (79)	6.06 (40)	5.07 (34)	3.57 (24)	3.30 (24)	1.73 (13)	2.69 (18)
5	16.78 (151)	12.68 (107)	11.05 (87)	7.39 (53)	7.83 (58)	5.58 (37)	3.95 (25)	3.39 (22)	1.76 (11)	1.81 (13)	1.60 (10)	1.37 (11)
6	11.44 (101)	8.45 (75)	7.18 (60)	5.12 (42)	3.64 (28)	3.76 (27)	3.18 (20)	1.85 (11)	1.26 (10)	1.39 (9)	1.91 (12)	0.62 (5)
7	8.19 (73)	9.14 (79)	5.13 (44)	2.85 (25)	2.98 (23)	2.42 (16)	2.03 (14)	1.16 (7)	0.99 (7)	0.77 (5)	0.60 (4)	0.77 (5)
8	5.77 (52)	7.62 (66)	2.81 (25)	1.68 (15)	1.86 (15)	2.70 (21)	2.11 (14)	0.93 (7)	0.49 (3)	0.49 (3)	1.19 (8)	1.17 (8)
9	4.36 (41)	3.18 (29)	1.77 (17)	1.72 (15)	1.92 (16)	0.99 (8)	0.71 (5)	0.60 (4)	0.91 (6)	0.95 (7)	0.47 (3)	0.73 (5)
10	3.75 (35)	2.46 (27)	1.14 (10)	1.19 (10)	0.49 (4)	0.96 (9)	0.74 (6)	0.28 (2)	0.74 (5)	0.90 (6)	0.63 (4)	0.00 (0)
11	2.58 (24)	1.55 (15)	1.69 (15)	1.38 (12)	1.07 (9)	0.99 (8)	0.48 (4)	0.74 (7)	0.56 (4)	0.58 (5)	0.59 (4)	0.61 (4)
12	1.72 (16)	0.45 (5)	0.67 (6)	1.36 (12)	0.92 (8)	0.48 (4)	0.25 (2)	0.36 (3)	0.37 (4)	0.27 (2)	0.14 (1)	0.15 (1)
13	0.95 (8)	0.81 (9)	0.57 (5)	0.34 (3)	0.46 (5)	0.58 (5)	1.07 (10)	0.12 (1)	0.36 (4)	0.36 (3)	1.37 (10)	0.86 (7)

Values are presented as CIR (number).

1 1,000 person.

## SUPPLEMENTARY MATERIALS

Supplementary Material: Korean version is available at http://www.e-epih.org/).
