# Supplementary material for: Incidence of varicella in children in Jeju-do, Korea, 2005-2016: age-period-cohort analysis
Source: Epidemiol Health. 2018 Nov 8;40:e2018054. doi: 10.4178/epih.e2018054 (PMC6302224; doi:10.4178/epih.e2018054)
Supplement: Supplementary file 1 [file epih-40-e2018054-supplementary.pdf]

Volume: 40, Article ID: e2018054, 7 pages

https://doi.org/10.4178/epih.e2018054

<Original Article>

제주도 소아의 수두발생, 2005-2016: 연령-기간-코호트 분석

Incidences of Varicella in Children of Jeju-do, Republic of Korea, 2005-2016: Age-Period-Cohort Analysis

김진희[1)], 김지은[1)], 배종면[1,2),]

1) 제주감염병관리지원단; 2) 국립제주대학교 의학전문대학원 예방의학교실

Corresponding authors: Jong-Myon Bae

jmbae@jejunu.ac.kr

064-755-5567

Volume: 40, Article ID: e2018054, 7 pages

https://doi.org/10.4178/epih.e2018054

Abstract

Objectives: Although the nationwide inoculation rate of varicella vaccine was approximately 95% in Korean children recently, the number of notified varicella cases is unexpectedly continuously increasing till now. To suggest some hypotheses regarding this discrepancy, an age-period-cohort (APC) analysis as a descriptive epidemiology study was conducted for children residing in Jeju-do, Republic of Korea.

Methods: The raw data were obtained from the nationwide database for insurance claim of healthcare fee provided by the National Health Insurance Service, Republic of Korea. The selection criteria were children aged 2–13 years who visited any healthcare center due to varicella from 2005 to 2016 while residing in Jeju-do. After calculating the birth cohort-specific crude incidence rates by age and year, the intrinsic estimator method was used to perform the APC analysis.

Results: As the annual crude incidence rates decreased with increasing age between 2005 and 2016, the age and period effects also decreased. The intrinsic estimator coefficients suggesting the cohort effect shifted from positive to negative in 2011, the starting year of free varicella vaccine program in Jeju-do.

Conclusions: The results suggested that inoculated varicella vaccines have preventive effects. However, further studies to evaluate waning immunity would be needed.

Keyword: Chickenpox, Chickenpox vaccine, Cohort effect, Immunization programs, Immunization schedule, Measles-Mumps-Rubella vaccine

Volume: 40, Article ID: e2018054, 7 pages

https://doi.org/10.4178/epih.e2018054

서론

1998년 세계보건기구 (WHO)가 수두 예방 접종을 권유한 것을 계기로 [1], 한국은 2005년 1월에 수두 예방접종이 국가필수 예방접종으로 지정하고 12-15개월의 영유아에게 1회 접종을 시행하고 있다 [2]. 국가통계포털 (Koran Statistical Information Service, http://kosis.kr) 에서 2016년도 수두백신의 전국 접종률은 97.5%에 달하였다 [3]. 시판전 임상시험 결과 한 번의 수두 백신 접종을 받을 경우 95%에서 면역력을 갖는 항체가 생성되며 [4], 시판 후 백신효과 (vaccine effectiveness, VE %)은 80-85% 수준으로 알려져 있다 [5-6]. 또한 백신 접종 후 10년 추적한 결과 1회 접종으로 94.4%에서 VE가 유지됨을 발표하였다 [7].

이렇게 큰 VE와 높은 접종률에 따라 국내 수두 환자 발생이 크게 저하하기를 기대할 수 있음에도 불구하고, 질병관리본부가 운영하는 감염병웹통계시스템의 수두 발생신고 건수는 2007년 20,284건에서 2016년 54,060건으로 10년간 2.7배 증가하였고 [2], 접종력을 가진 아이에서 돌발수두 (breakthrough varicella)가 주로 발생하고 있다고 보고하고 있다 [8-10]. 2011년 이후 생후 12-15개월 유아에게 수두백신 무상접종을 시행해 오고 있는 제주도에 있어서, 2015-2016년도 수두백신 접종률이 97.0%에 달함에도 불구하고 [3], 수두발생 건수가 2017년도 같은 기간 대비하여 2018년도는 125% 증가하였으며 [11], 수두역학조사

에서 발생 환자 모두 백신 접종력이 있는 것으로 보고되었다 [12].

이렇게 수두백신접종사업을 수행하지만 환자 발생이 많아지는 괴리 현상에 있어, 타당한 해설을 하기 위해서는 기술역학연구가 필요하다. 따라서 제주도 아동의 수두발생에 있어 연령-기간-코호트 (age-period-cohort, APC) 분석을 하여 가설을 제시하는 것이 본 연구의 목적이다.

연구대상 및 방법

본 연구의 원자료는 (raw data) 국민건강보험공단 (National Health Insurance Service, NHIS, http://www.nhis.or.kr)이 제공하는 건강보험 빅데이터 맞춤형연구 DB이다. 이는 전 국민 중 국내 의료기관에서 진료를 받을 경우, 해당 의료기관이 NHIS에 수진 청구를 하면서 만들어진 데이터베이스로 전수자료에 해당한다.

대상자 선정기준은 (1) 2005년도에서 2016년도에 걸쳐 수진청구 시 주상병과 9개 부상병 질병코드가 한국표준질병분류 기준 B01~B01.9까지로 입력된 사례로 (2) 주소지가

Volume: 40, Article ID: e2018054, 7 pages

https://doi.org/10.4178/epih.e2018054

제주도로 되어 있으면서 (3) 청구 당시 연령이 만 2-13세이다. 관찰 연도시작을 2005년으로 한 것은 수두 예방접종을 국가 필수 예방접종으로 지정한 연도를 감안한 것이다. 연령을 2세 이상으로 한 것은 생후 12-15개월에 접종하도록 되어 있는 국내 수두백신 접종지침을 고려한 것이며, 13세 이하로 정한 것은, 수두 발생이 초등학생들 이하에서 발생한 것을 고려한 것이다. 그리고 한 대상자에서 1년 이내에 수두 질병코드가 여러 번 청구된 경우는, 과거의 수두 감염에 의한 연속 치료로 간주하고 제외시켰다.

연도별, 연령별 수두 조발생률 (crude incidence rate, CIR; per 1,000 person)은 구축된 자료에서 얻어낸 동일 연령과 동일 연도의 수진 건수를 분자로 삼고, 통계청 (http://kosis.kr)이 제공하는 동일연령, 동일 연도의 연앙인구수를 분모로 삼아서 산출한 수치로 정의하였다.

APC 분석을 위해 Intrinsic Estimator (IE) 법을 적용하였다 [13]. 연령과 기간에 따라 코호트가 결정되기에, 연령-기간-코호트 간의 선형적 의존성 (linear dependency)을 해결하기 위해 개발된 통계법이기 때문이다 [14]. IE 적용을 위해 Stata SE ver. 14 (www.stata.com)가 제공하는 <*apc_ie*> 패키지를 이용하였다. 본 연구는 개인정보를 모르는 2차 자료의 활용인 점에서 소속 대학교의 연구윤리심의위원회 심의 면제를 받았다.

Volume: 40, Article ID: e2018054, 7 pages

https://doi.org/10.4178/epih.e2018054

연구결과

표 1은 2005년도에서 2016년도까지 2-13세 소아의 수두 CIR을 제시한 것이다. 모든 연도에서 연령이 증가할수록 CIR은 감소하였다. 무상으로 1차 접종이 제공되기 전인 2005-2010년 구간에서는 모든 연령에서 연도가 최근으로 올수록 CIR은 감소하는 흐름을 보였다. 2011년을 지나면서 2세-6세군은 감소 경향을 여전히 유지하는 반면, 7세군 이상에서는 감소 흐름이 사라지거나 증가하는 양상을 보였다. 그림 1은 표의 자료를 연령별로 출생코호트에 따른 CIR을 그래프로 제시한 것이다. 동일 연령군에서 출생코호트가 최근으로 올수록 감소하는 경향을 보였다.

그림 2는 2005년도에서 2016년도까지 2-13세 소아의 수두 CIR에 대한 연령, 기간, 코호트 효과를 분석한 결과이다. 앞서 해석을 뒷받침하듯이 기간효과와 코호트 효과를 보정한 상태에서 연령이 증가할수록 수두 발생률은 감소하였으며, 연도별로도 연령효과와 코호트 효과를 보정한 상태에서 최근으로 올수록 수두 발생률은 감소하였다. 또한 연령효과와 기간효과를 보정한 상태에서 코호트 효과는 2012년 이후 출생한 아이에서 수두발생 위험이 감소하는 추세를 보였다.

그림 3은 제주도에서 수두백신 무상접종이 시작된 2011년 이전인 2005-2010년도의

수두 CIR에 대한 APC 분석결과이다. 연령이 증가할수록 수두 발생률이 감소하는 추세를 보였고, 연도가 2005년에서 2008년으로 오면서 감소하다가 정체하는 추세를 보였다. 반면 출생연도가 1992년에서 2008년으로 이동할수록 수두 발생률은 지속적으로 증가하는 추세를 보였다.

그림 4은 제주도에서 수두백신 무상접종이 시작된 2011년부터 2016년도의 수두 CIR에 대한 APC 분석결과이다. 연령이 증가할수록 수두 발생률이 감소하는 추세를 보였고, 연도가 2011년에서 2016년으로 오면서 감소하는 추세를 보였다. 츨생년도가 2012년 이후 감소하는 추세를 보였다.

토론

APC 분석 결과들을 요약하자면, 연령이 증가하고 연도가 최근으로 올수록 수두 발생 위험도가 감소하였으며, 수두백신 무상접종이 시작된 2011년 이후 출생한 군에서 수두 발생 위험도가 감소하였다. 다시 말해서, NHIS 자료에 근거할 때 2005년부터 2016년도까지 제주도 아동의 수두 발생률은 감소하였으며, 이는 수두예방백신의 효과가 있음을 지지하는 결과이다.

Volume: 40, Article ID: e2018054, 7 pages

https://doi.org/10.4178/epih.e2018054

이상의 수두 백신의 예방효과가 있다는 결과에 따른다면, 몇 가지 점들을 추론해 볼 수 있다. 첫째, 예방접종 지침에 따라 지속적으로 백신접종사업을 유지하고 높은 접종률을 달성해 나간다면, 2011년도 출생한 아동이 14세가 되는 2025년 이후로는 수두 발생은 감소할 것으로 예측할 수 있다. 이는 연령이 증가할수록 수두발생에 미치는 효과는 감소한다는 결과를 전제한 것이다. 둘째, 수두 백신을 접종한 이후에도 수두 발생이 증가하는 현상에 대하여 백신의 효과와 접종실패를 우려하는 상황에서, 제주도 아동에게 접종해온 수두 백신은 감염 예방의 효과가 있음을 지지하는 근거가 된다. 이는 무상백신접종이 시작된 2011년 이후 5년간의 자료를 분석한 APC 결과를 전제한 것이다.

그러나 수두 백신의 예방효과가 있다는 근거에도 불구하고, 제주도의 수두환자 발생신고건수는 지속적으로 증가하는 상황에 대하여는 다음과 같은 추론을 해 볼 수 있다. 첫째, 본 연구의 자료원인 NHIS 수진청구 건수와 질병관리본부의 수두감시 연보 신고건수와의 괴리에 따른 것으로 해석할 수 있다. 두 자료원간의 괴리는 과거로 갈수록 심하고, 최근에 올수록 줄어드는 양상을 보이고 있기 때문이다 [2]. 따라서 질병관리본부의 수두 신고건수에 따르면 급격히 증가하고 있지만, NHIS 수진청구 건수에 따르면 감소하여 왔다고 해석할 수 있는 것이다. 둘째, 2018년 현재 제주도 아동의 수두 발생건수 증가는 무상접종이 시작된 2011년 이전에 출생한 아동군에서 상대적으로 높은 발생률에 기인한 것으로 해석할

Volume: 40, Article ID: e2018054, 7 pages

https://doi.org/10.4178/epih.e2018054

수 있다. 이는 2011년 이후 현재까지 국가예방접종 지침에 따라 생후 12-15개월에 1회 의무접종을 하고 있고 접종률이 97%를 상회함을 전제한 것이다.

또한 수두 백신의 예방효과가 있다는 근거에도 불구하고, 수두접종력이 있는 아이에서 돌발수두가 발생하는 사례들 [8-12]에 대하여는 합당한 해석을 위한 가설제시가 필요하다. 돌발수두 발생은 접종 후 면역력 획득이 안 된 1차 백신실패 (primary vaccine failure) 보다는, 생후 12-15개월에 접종한 수두백신이 시간이 경과하면서 획득한 면역력을 유지하지 못한 2차 백신 실패 (secondary vaccine failure) 로 해석할 수 있는데, 그 관련 근거들은 다음과 같다 [1]. 첫째, 단면조사에 의한 혈청역학연구로 국내 2009-2010년 수집한 혈청에서 수두 바이러스의 혈청 양성률 (seroprevalence)에서 1-2세 75% 수준에서 3-5세 57.5%로 떨어졌다 [15]. 둘째, 환자-대조군 연구로 생후 15개월 이전에 수두접종을 했던 5세 국내 아동에서 수두 예방 접종의 VE가 54% [8], 13% (95% CI: -17.3~35.6) [16]라는 보고이다. 셋째, 미국 아동에 대한 코호트 추적결과 접종 후 시간이 지날수록 돌발수두의 발생률이 높아진다는 것이다 [17]. 이에 따라 미국은 2006년 이후 4-6세에 2차 접종을 하고 있다 [18,19].

이처럼 2차 백신 실패로 2차 접종의 필요성이 대두되는 현상에 대한 실마리를 제공하는 연구결과가 있다. 2003년 발표된 후향적 코호트 연구에서 [20] 생백신인 홍역-볼거리-

Volume: 40, Article ID: e2018054, 7 pages

https://doi.org/10.4178/epih.e2018054

풍진 (Measles-Mumps-Rubella, MMR) 백신 접종 후 28일 이내에 수두 백신 접종을 했을 때, 돌발수두 발생 위험이 3.1 배 (95% CI: 1.5-6.4) 높아진다고 보고하였다. 이는 두 가지 다른 생백신간의 면역획득과정의 간섭 (interference)을 줄이기 위해서 가급적 4주 간격으로 접종한다는 백신접종의 원칙에 어긋나서는 안된다는 근거가 된다 [2]. 그런데, 해당 연구에서 동시접종에 대한 추가 자료가 없지만 연구방법에서 간격 산출에 대한 서술로 볼 때, 간격이 0일인 동시접종군도 돌발수두 위험군에 포함되었을 개연성이 높다.

2009년 이후 홍역-볼거리-풍진-수두 (Measles-Mumps-Rubella-Varicella, MMRV) 백신제가 두 백신의 동시접종 (MMR+V)에 비하여 면역획득력 (immunogenicity)과 효능 (efficacy) 측면의 비열등성 (non-inferior)의 관련 근거에 따라 시판 허가가 났으며 [21], 현재 미국, 독일, 호주, 이태리, 인도 등에서 접종되고 있다 [20-24]. 국내 예방접종 지침에서도 예방접종률을 높이고 적기에 예방접종을 받을 수 있다는 장점에 따라, MMR 백신 1차 접종시기와 수두 백신의 접종을 같은 날 동시에 접종(MMR+V)을 권하고 있다 [2]. 요약하자면 3 접종법 - MMR+V 동시접종, V 접종후 4주 지나서 MMR 순차접종 (V>MMR), 그리고 MMR 접종후 4주 지나서 V 순차접종 (MMR>V) - 간의 장기간 백신효과를 직접 비교(head-to-head)하는 비교효과연구 (comparative effectiveness research)가 없이 접종현장에서는 동시접종 중심으로 예방사업이 이루어 지고 있는 것이다.

Volume: 40, Article ID: e2018054, 7 pages

https://doi.org/10.4178/epih.e2018054

그런데, 홍역 바이러스 감염시 세포면역 억제 효과가 있다고 알려져 있다. 실험실 연구에서 홍역 감염시 단핵구 (mononuclear cell)에서 생산하는 interleukin 과 interferon 이 낮은 수준을 보이고, 지연된 과민반응이 없는 등 세포면역체계의 저하가 여러 주 (several weeks)에 걸쳐 나타나는 것으로 보고되었으며 [25-28], 이러한 세포면역의 이상반응은 홍역 백신 접종 후에도 일시적으로 나타난다고 보고하였다 [28-30]. 그렇다면 현재의 MMR+V 동시접종과 V>MMR 순차접종 간의 장기적 백신효과에 대한 연구가 필요하다. 이에 대한 근거를 얻기 위해서는, 국가예방접종 지침을 벗어나지 않는 범위에서 12-13개월에 수두 백신을 우선 접종하고, 4주를 넘긴 다음 14-15개월에 MMR 백신 1차 접종을 하는 순차접종법을 도입하는 것이다. 그래서 동시접종군과 순차접종군에 대한 전향적 코호트 연구를 통해 돌발수두의 발생 위험도를 알아 내어야 한다. 만약 V>MMR 순차접종법으로 수두 면역 획득력이 동시접종법보다 더 향상된다는 결과가 나올 경우 2차 접종의 필요성이 없어질 수 있겠다. 따라서, 국내 아동들에 있어서 수두백신의 접종방법별 수두 백신의 장기적 예방효과에 대한 다각도의 역학연구가 필요하다.

본 연구의 한계점은 다음과 같다. 첫째, 수두 감염에 대한 혈청학적 검사에 기반한 확진이 아닌 진료수진 청구자료를 활용했기에, 수두 의심환자도 포함될 수 있다는 점이다. 그러나 수두의 진단은 실험실적 검사보다는 의료진의 임상적 진찰로 이루어지는 것을 감안한다면, 수진자료의 활용에 대한 타당성은 있다고 본다. 둘째, 건수 단위로 자료를 얻었기에

Volume: 40, Article ID: e2018054, 7 pages

https://doi.org/10.4178/epih.e2018054

사례별 돌발수두 여부를 구별할 수 없다는 점이다. 그러나 무상백신접종이 시작된 2011년 이후 출생아에 있어 높은 접종률을 반영할 때, 수두 청구건수는 대부분 돌발수두로 볼 수 있겠다. 다만 2010년 이전의 출생아에 대하여는 이를 감안해서 해석할 필요가 있다. 셋째, 돌발수두로 발현되는 임상증상이 미약한 경우는 의료기관을 방문하지 않을 수 있겠다. 그렇다면 본 자료의 CIR은 실제보다 과소추정 (under-estimated)되었다고 볼 수 있다. 다만 이 개연성의 확률이 연령별, 연도별로 차이가 없다고 본다면 APC 결과에 대한 해석은 여전히 유효하겠다. 넷째, 수두 발생 환자를 정의할 때 건강보험공단의 수진자료에서 주상병 및 부상병 9순위까지 포함했기에, 해당 선정 기준을 어떻게 정하는가에 따라 CIR 산출치가 차이 날 수 있다. 그러나 본 연구는 CIR의 절대값 산출보다는 연령-기간-코호트 효과를 분석하는 것을 목적으로 하기에, 환자 기준의 일관성을 부여하는 것이 더 중요하겠다. 향후 건강보험공단에서 수두 환자의 타당한 기준을 설정하는 민감도 분석이 필요하겠다.

결론적으로 수두가 법정관리감염병으로 지정되고 백신접종이 권장된 2005년 이후 최근 연도로 오면서 수두 발생률이 감소하는 기간 효과와, 제주도에서 무상접종을 시작한 2011년 이후 출생자에서 수두발생률 위험이 감소한 코호트 효과를 확인하였다. 따라서, 현재 접종하고 있는 수두 백신은 수두발생을 예방하는 효과가 있다고 해석할 수 있다. 다만 장기적 효과에 대한 근거확보를 위해 접종방법에 따른 비교효과연구가 필요하며, 이를 위해

Volume: 40, Article ID: e2018054, 7 pages

https://doi.org/10.4178/epih.e2018054

선 12개월 영아에게 수두 접종을 한 뒤 4주후에 MMR 접종을 하는 순차접종법을 도입하기 를 제안한다.

Volume: 40, Article ID: e2018054, 7 pages

https://doi.org/10.4178/epih.e2018054

References

1. Papaloukas O, Giannouli G, Papaevangelou V. Successes and challenges in varicella vaccine. Ther Adv Vaccines. 2014;2(2):39-55.

2. KCDC. The guideline for controlling vaccine-preventable infectious diseases 2018. [cited 2018 Nov 7]. Available from: https://nip.cdc.go.kr/irgd/reference.do

3. KOSIS. Statistics for innoculation rate in varicella vaccine. [cited 2018 Sept 24]. Available from: http://kosis.kr/statHtml/statHtml.do?orgId=117&tblId=DT_11793N_151&vw_cd=MT_ZTITLE&list_id=D1_A01_A02&seqNo=&lang_mode=ko&language=kor&obj_var_id=&itm_id=&conn_path=E1#

4. Provost PJ, Krah DL, Kuter BJ, Morton DH, Schofield TL, Wasmuth EH, et al. Antibody assays suitable for assessing immune responses to live varicella vaccine. Vaccine. 1991;9(2):111-6.

5. World Health Organization. Systematic review of available evidence on effectiveness and duration of protection of varicella vaccines. [cited 2018 Nov 7]. Available from: http://www.who.int/immunization/sage/meetings/2014/april/presentations_background_docs/en/

6. Marin M, Marti M, Kambhampati A, Jeram SM, Seward JF. Global varicella vaccine effectiveness: a meta-analysis. Pediatrics. 2016;137:e20153741.

7. Kuter B, Mattews H, Shinefield H, Black S, Dennehy P, Watson B, Reisinger K, et al. Ten year follow-up of healthy children who received one or two injections of varicella vaccine. Pediatr Infect Dis J. 2004;23:132-37.

8. Oh SH, Choi EH, Shin SH, Kim YK, Chang JK, Choi KM, et al. Varicella and varicella vaccination in South Korea. Clin Vaccine Immunol. 2014;21(5):762-8.

9. Park CS, Kim DS, Kim KH. Varicella outbreak in the patients during group therapy: seroprevalence in a healthcare system during breakthrough varicella occurrence. Clin Exp Vaccine Res. 2013;2(2):140-3.

10. Park CK, Kim HO, Park CW, Lee CH. The effect of active immunization on the clinical courses of varicella. Korean J Dermatol. 2008;46(8):1011-1019. (Korean)

11. Jeju Center for Infection Control. The seasonal report of infectious diseases on Spring (March, April, May) in 2018, Jeju-do. [cited 2018 Sept 24]. Available from: http://jeci.kr/bbs/board.php?bo_table=weekreport&wr_id=19 (Korean)

12. Oh HS, Bae JM. Vaccination history in elementary school children enrolled in the varicella epidemic investigations held in Jeju-si, Korea in the first half of 2017. Epidemiol Health. 2017;39:e2017053.

13. Yang Y, Fu WJ, Land KC. The intrinsic estimator for age-period-cohort analysis: What it is and how to use it. Am J Sociol. 2008;113(6):1697-1736.

14. Clayton D, Shifflers E. Models for temporal variation in cancer rates. II: age-period-cohort models. Stat Med. 1987;6(4):469-81.

15. Lee H, Cho HK, Kim KH. Seroepidemiology of varicella-zoster virus in Korea. J Korean Med Sci. 2013;28(2):195-9.

16. Lee YH, Choe YJ, Cho SI, Kang CR, Bang JH, Oh MD, et al. Effectiveness of Varicella Vaccination Program in Preventing Laboratory-Confirmed Cases in Children in Seoul, Korea. J Korean Med Sci. 2016;31(12):1897-1901.

17. Chaves SS, Gargiullo P, Zhang JX, Civen R, Guris D, Mascola L, et al. Loss of vaccine-induced immunity to varicella over time. N Engl J Med. 2007;356(11):1121-9.

18. Marin M, Güris D, Chaves SS, Schmid S, Seward JF; Advisory Committee on Immunization Practices, Centers for Disease Control and Prevention (CDC). Prevention of varicella: recommendations of the Advisory Committee on Immunization Practices (ACIP). MMWR Recomm Rep. 2007;56(RR-4):1-40.

19. Lopez AS, Zhang J, Marin M. Epidemiology of Varicella During the 2-Dose Varicella Vaccination Program - United States, 2005-2014. MMWR Morb Mortal Wkly Rep. 2016;65(34):902-5.

20. Verstraeten T, Jumaan AO, Mullooly JP, Seward JF, Izurieta HS, DeStefano F, et al. A retrospective cohort study of the association of varicella vaccine failure with asthma, steroid use, age at vaccination, and measles-mumps-rubella vaccination. Pediatrics. 2003;112(2):e98-103.

21. Marin M, Broder KR, Temte JL, Snider DE, Seward JF; Centers for Disease Control and Prevention (CDC). Use of combination measles, mumps, rubella, and varicella vaccine: recommendations of the Advisory Committee on Immunization Practices (ACIP). MMWR Recomm Rep. 2010;59(RR-3):1-12.

22. Giaquinto C, Gabutti G, Baldo V, Villa M, Tramontan L, Raccanello N, et al. Impact of a vaccination programme in children vaccinated with ProQuad, and ProQuad-specific effectiveness against varicella in the Veneto region of Italy. BMC Infect Dis. 2018;18(1):103.

23. Shah N, Parikh R, Casabona G, Kolhapure S. A New Combined Vaccine Against Measles, Mumps, Rubella and Varicella in India. Indian Pediatr. 2017;54(12):1041-1046.

24. Macartney K, Gidding HF, Trinh L, Wang H, Dey A, Hull B, et al. Evaluation of Combination Measles-Mumps-Rubella-Varicella Vaccine Introduction in Australia. JAMA Pediatr. 2017;171(10):992-998.

25. Griffin DE. Immune responses during measles virus infection. Curr Top Microbiol Immunol.

1995;191:117-34.

26. Moss WJ, Ota MO, Griffin DE. Measles: immune suppression and immune responses. Int J Biochem Cell Biol. 2004;36(8):1380-5.

27. Kerdiles YM, Sellin CI, Druelle J, Horvat B. Immunosuppression caused by measles virus: role of viral proteins. Rev Med Virol. 2006;16(1):49-63.

28. Karp CL, Wysocka M, Wahl LM, Ahearn JM, Cuomo PJ, Sherry B, et al. Mechanism of suppression of cell-mediated immunity by measles virus. Science. 1996;273(5272):228-31.

29. Ward BJ, Griffin DE. Changes in cytokine production after measles virus vaccination: predominant production of IL-4 suggests induction of a Th2 response. Clin Immunol Immunopathol. 1993;67(2):171-7.

30. Gans HA, Yasukawa LL, Zhang CZ, Wakim RH, Rinki M, Dehovitz R, et al. Effects of interleukin-12 and interleukin-15 on measles-specific T-cell responses in vaccinated infants. Viral Immunol. 2008;21(2):163-72.

Volume: 40, Article ID: e2018054, 7 pages

https://doi.org/10.4178/epih.e2018054

Table1. Annual age-specific crude incidence rates (CIR, 1000 person) and numbers (N) of varicella cases by age (2-13 years old) and calendar year (2005-2016) in children of Jeju-do, Republic of Korea*

**Age**	**Calendar year**											
	**2005**	**2006**	**2007**	**2008**	**2009**	**2010**	**2011**	**2012**	**2013**	**2014**	**2015**	**2016**
**2**	95.55 (664)	69.69 (453)	45.31 (294)	26.60 (167)	25.37 (166)	27.36 (177)	24.61 (148)	13.85 (82)	9.62 (62)	9.52 (68)	7.17 (48)	5.86 (41)
**3**	53.23 (400)	36.25 (247)	21.09 (147)	15.69 (104)	16.79 (103)	16.86 (113)	11.10 (70)	7.66 (47)	7.19 (43)	3.27 (23)	4.67 (33)	3.91 (28)
**4**	25.23 (216)	20.54 (155)	13.44 (97)	9.55 (67)	11.35 (76)	12.46 (79)	6.06 (40)	5.07 (34)	3.57 (24)	3.30 (24)	1.73 (13)	2.69 (18)
**5**	16.78 (151)	12.68 (107)	11.05 (87)	7.39 (53)	7.83 (58)	5.58 (37)	3.95 (25)	3.39 (22)	1.76 (11)	1.81 (13)	1.60 (10)	1.37 (11)
**6**	11.44 (101)	8.45 (75)	7.18 (60)	5.12 (42)	3.64 (28)	3.76 (27)	3.18 (20)	1.85 (11)	1.26 (10)	1.39 (9)	1.91 (12)	0.62 (5)
**7**	8.19 (73)	9.14 (79)	5.13 (44)	2.85 (25)	2.98 (23)	2.42 (16)	2.03 (14)	1.16 (7)	0.99 (7)	0.77 (5)	0.60 (4)	0.77 (5)

Volume: 40, Article ID: e2018054, 7 pages

https://doi.org/10.4178/epih.e2018054

**8**	5.77 (52)	7.62 (66)	2.81 (25)	1.68 (15)	1.86 (15)	2.70 (21)	2.11 (14)	0.93 (7)	0.49 (3)	0.49 (3)	1.19 (8)	1.17 (8)
**9**	4.36 (41)	3.18 (29)	1.77 (17)	1.72 (15)	1.92 (16)	0.99 (8)	0.71 (5)	0.60 (4)	0.91 (6)	0.95 (7)	0.47 (3)	0.73 (5)
**10**	3.75 (35)	2.46 (27)	1.14 (10)	1.19 (10)	0.49 (4)	0.96 (9)	0.74 (6)	0.28 (2)	0.74 (5)	0.90 (6)	0.63 (4)	0.00 (0)
**11**	2.58 (24)	1.55 (15)	1.69 (15)	1.38 (12)	1.07 (9)	0.99 (8)	0.48 (4)	0.74 (7)	0.56 (4)	0.58 (5)	0.59 (4)	0.61 (4)
**12**	1.72 (16)	0.45 (5)	0.67 (6)	1.36 (12)	0.92 (8)	0.48 (4)	0.25 (2)	0.36 (3)	0.37 (4)	0.27 (2)	0.14 (1)	0.15 (1)
**13**	0.95 (8)	0.81 (9)	0.57 (5)	0.34 (3)	0.46 (5)	0.58 (5)	1.07 (10)	0.12 (1)	0.36 (4)	0.36 (3)	1.37 (10)	0.86 (7)

* CIR (N)

Volume: 40, Article ID: e2018054, 7 pages

https://doi.org/10.4178/epih.e2018054
